# Sequencing of emerging canine distemper virus strain reveals new distinct genetic lineage in the United States associated with disease in wildlife and domestic canine populations

**DOI:** 10.1186/s12985-015-0445-7

**Published:** 2015-12-18

**Authors:** Matthew C. Riley, Rebecca P. Wilkes

**Affiliations:** Department of Biomedical and Diagnostic Sciences, College of Veterinary Medicine, The University of Tennessee, 2407 River Drive, 37996 Knoxville, TN USA; United States Army, Medical Service Corps, ᅟ, USA; Present address: Veterinary Diagnostic and Investigational Laboratory, College of Veterinary Medicine, The University of Georgia, 43 Brighton Road, 31793 Tifton, GA USA

**Keywords:** Canine distemper virus, CDV, Genome sequencing, New CDV strain, Phylogenetics, Vaccination, Vaccines

## Abstract

**Background:**

Recent outbreaks of canine distemper have prompted examination of strains from clinical samples submitted to the University of Tennessee College of Veterinary Medicine (UTCVM) Clinical Virology Lab. We previously described a new strain of CDV that significantly diverged from all genotypes reported to date including America 2, the genotype proposed to be the main lineage currently circulating in the US. The aim of this study was to determine when this new strain appeared and how widespread it is in animal populations, given that it has also been detected in fully vaccinated adult dogs. Additionally, we sequenced complete viral genomes to characterize the strain and determine if variation is confined to known variable regions of the genome or if the changes are also present in more conserved regions.

**Methods:**

Archived clinical samples were genotyped using real-time RT-PCR amplification and sequencing. The genomes of two unrelated viruses from a dog and fox each from a different state were sequenced and aligned with previously published genomes. Phylogenetic analysis was performed using coding, non-coding and genome-length sequences. Virus neutralization assays were used to evaluate potential antigenic differences between this strain and a vaccine strain and mixed ANOVA test was used to compare the titers.

**Results:**

Genotyping revealed this strain first appeared in 2011 and was detected in dogs from multiple states in the Southeast region of the United States. It was the main strain detected among the clinical samples that were typed from 2011–2013, including wildlife submissions. Genome sequencing demonstrated that it is highly conserved within a new lineage and preliminary serologic testing showed significant differences in neutralizing antibody titers between this strain and the strain commonly used in vaccines.

**Conclusion:**

This new strain represents an emerging CDV in domestic dogs in the US, may be associated with a stable reservoir in the wildlife population, and could facilitate vaccine escape.

## Background

Canine distemper virus (CDV) is an enveloped negative-sense, single-stranded RNA virus that produces multi-systemic disease in dogs and other terrestrial carnivores [1]. Similar to measles virus, CDV is a member of the genus *Morbillivirus* within the family *Paramyxoviridae.* The virus is highly contagious and results in immunosuppression in the host. Clinical signs associated with infection may include gastrointestinal signs (vomiting and diarrhea) and/or respiratory signs that may be complicated by secondary bacterial infection (purulent nasal discharge, coughing, dyspnea, pneumonia). The infection may progress to the central nervous system (CNS) and may result in death.

Despite extensive vaccination of dogs in developed countries, recent reports suggest both re-emergence and increased activity of CDV worldwide, including the United States [2–6]. We have seen an increase in overall CDV positive samples submitted to University of Tennessee College of Veterinary Medicine (UTCVM) Clinical Virology Lab from 5 % in 2010 to 27 % in 2013 [7].

Traditionally, circulating field strains cluster into distinct clades. These distinct genotypes have been designated as genetic lineages and named based on the geographic regions where the genotypes were originally detected [2–4, 6]. While there have only been two North American lineages clearly defined, recent reports suggest at least 3 lineages (including 2 newly identified genotypes) are circulating in South America [3]. We previously sequenced regions from CDV positive samples and based on limited phylogenetic evaluation there appear to be 3–4 different “North American” genotypes currently circulating in the USA, including a new genotype of CDV we discovered circulating in Tennessee [7].

Most currently circulating strains are genetically divergent from the strains used for vaccine production. Onderstepoort and Snyder Hill strains (designated America 1 lineage) were used for vaccine production in the 1950s, and the vaccines have not changed since then [8, 9]. Many have questioned the efficacy of these vaccines with reports of CDV in previously vaccinated dogs since the 1990s [10], including recent reports in Japan [11], Mexico [12], Argentina [13], and the United States [5]. There are multiple reasons vaccines may fail aside from genetic differences, such as interference of maternal antibody resulting in incomplete protection and improper handling of the vaccine [1]. However, as newly emerging strains with significant genetic diversity from the available vaccine strains are detected, changes in virulence as well as antigenicity are anticipated in light of previously reported vaccine breakthroughs.

One aim of this present study was to evaluate additional archived samples to determine when this new strain first appeared, its geographic distribution, and if it is associated with the perceived increase in CDV cases from 2011 to 2013 and continuing into 2014. We then sought to investigate if this strain is conserved utilizing genome sequencing and phylogenetic analysis of two samples obtained from independent geographic regions, points in time and different species. We also performed initial serologic testing to evaluate presence of antigenic variation compared to a commonly used vaccine strain Onderstepoort. This type of information is necessary for future efforts toward determining vaccine efficacy.

## Results

Eighty-four of 352 samples tested from 2011 to 2013 were positive for CDV by real-time RT-PCR. Only one sample from each of 3 independent outbreaks in shelters and a pet store is included in these numbers, so the numbers actually do not reflect the total number of samples tested or the total number of positive samples. This was done to demonstrate that these positive samples were not just associated with isolated outbreaks. The majority of these samples were submitted from client-owned, young adult dogs from individual homes. Vaccination history was missing for several dogs, but most of the animals were unvaccinated or only partially vaccinated. Three adult dogs (ages 1, 4, and 8 years old) with complete vaccination histories were positive for CDV. Based on veterinary records, these dogs had been properly vaccinated according to vaccine guidelines and one of the dogs (8 year old) had actually been vaccinated yearly, rather than the recommended every three years schedule.

**Fig. 1:**
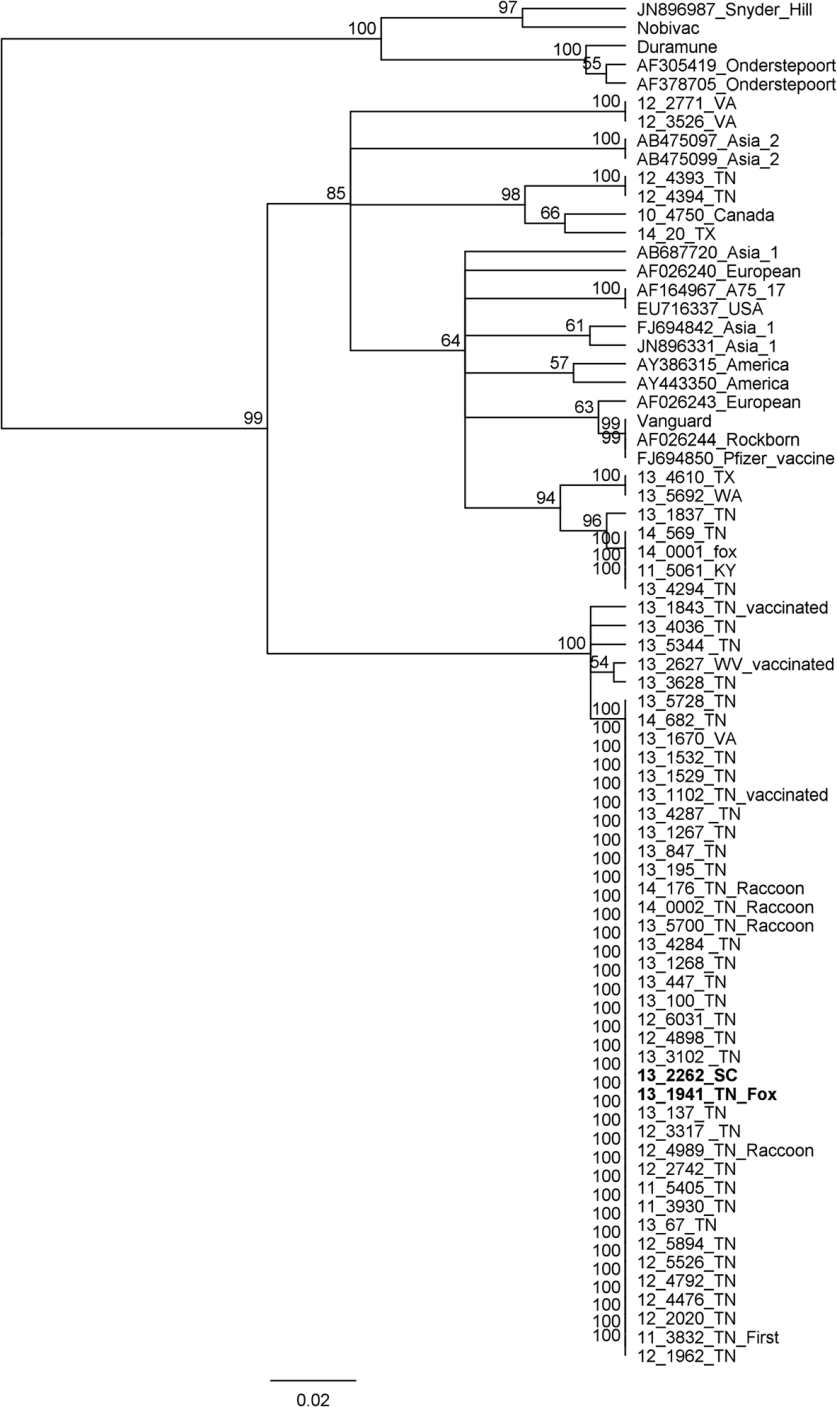
Phylogenetic tree comparing relatedness between M-F region of various CDV strains. The new strain described in this study represents a novel distinct clade significantly divergent from all previous sequences (lower branch). We also identified some sequences that match previously existing clades. Isolates used for genome sequencing are in bold, and their placement among the other isolates suggests that genome-level conservation is likely present in all isolates of the “new” strain. Consensus unrooted phylogenetic trees were generated within Geneious© using the Tamura-Nei model with UPGMA clustering and 1000 bootstrap replicates

Genotypes of 22 wild-type strains were previously reported [7] and are included in the numbers reported here for completeness. An additional 33 positive samples were typed for this report, including 5 samples from 2014. Genotyping of samples obtained from different geographic regions and different states revealed this new strain as the predominant strain from the majority of samples typed from 2011 to 2013 (75 % of the samples from 2011(3/4); 73 % from 2012(11/15); 86 % (24/28) of the samples from 2013), but it was not detected in samples collected in 2010 (0/5). This strain was detected in 35/39 samples typed from eastern Tennessee (between 2011 and 2013), including every county tested (10 different counties), and it was still circulating in Tennessee in 2014 (2/5). We also detected this novel strain in 1/3 samples typed from Virginia, 1/1 from South Carolina, and 1/1 from West Virginia. This strain was not detected in a limited number of samples from Canada, Washington, Texas, or Kentucky. These genotyping results using a portion of the M gene and the M-F region and compared to other published sequences are displayed as a phylogenetic tree (Fig. 1).

**Fig. 2:**
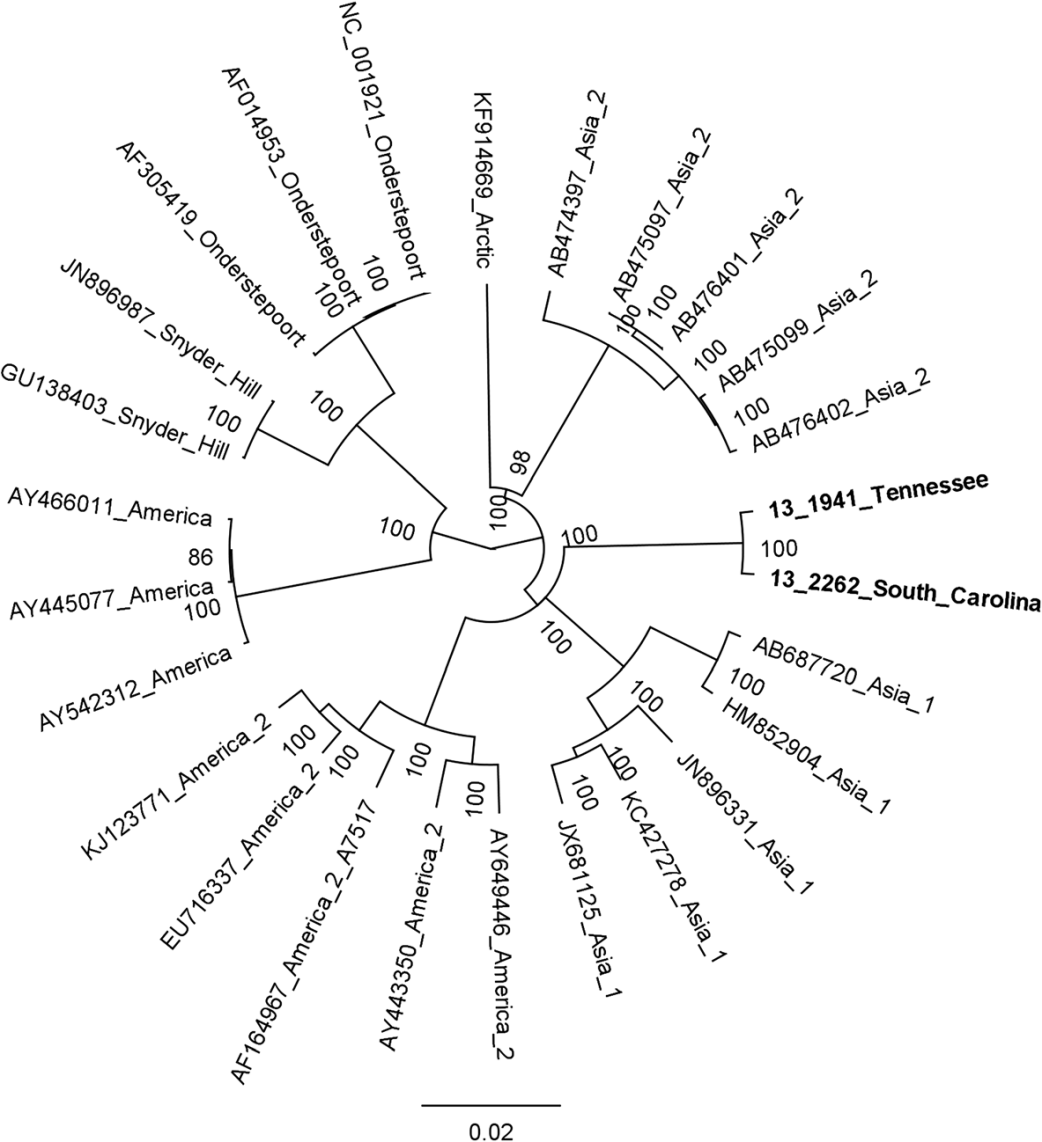
Phylogenetic tree for genome sequences representing major geographic regions. Two genomes sequenced from the new strain (bold) diverge significantly from other sequenced genomes. The distance between the new strain is similar to the distance between recognized geographically distinct strains, as well as vaccine strains. Tree was constructed using MrBayes (Bayesian inference) using the GTR substitution model with 1,000,000 iterations and subsampling every 1000 trees

**Fig. 3:**
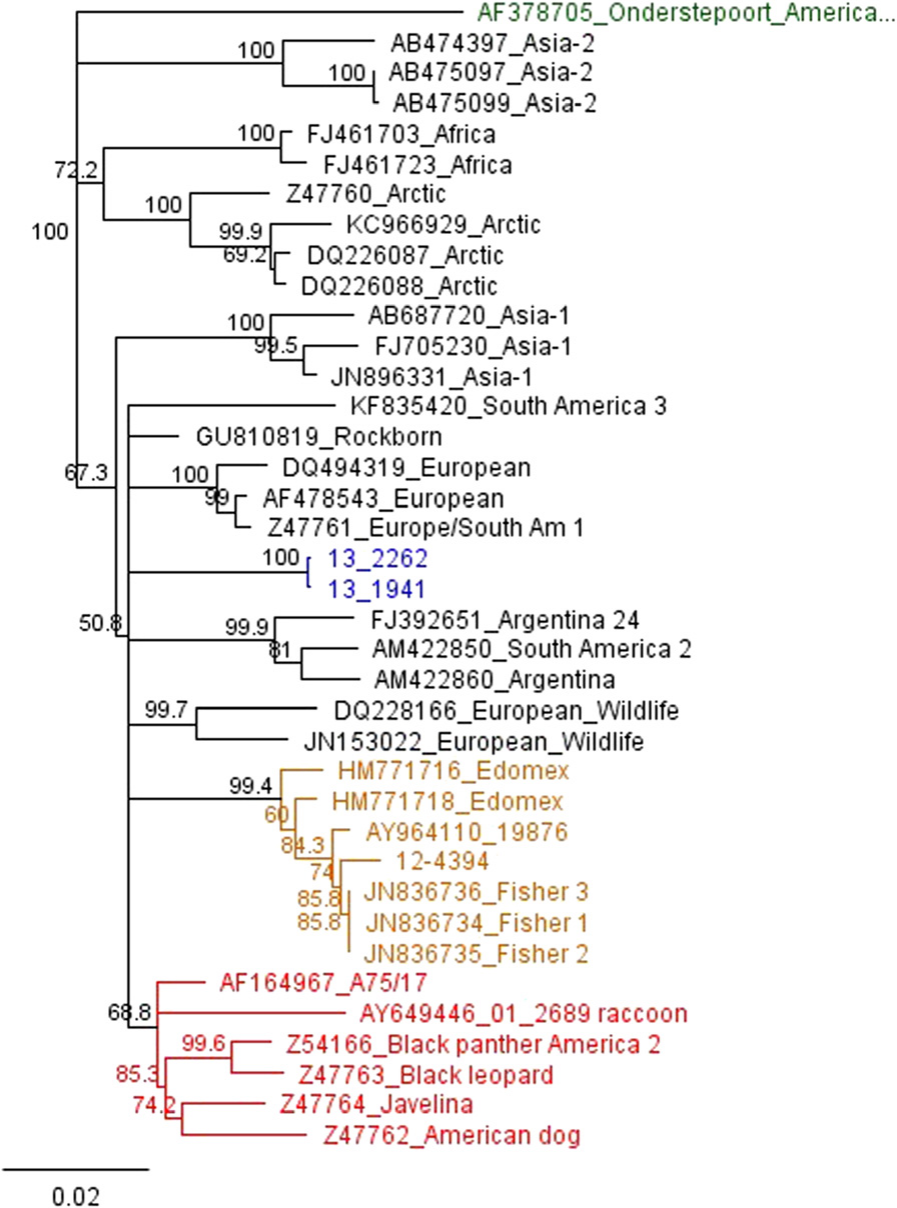
Phylogenetic tree for H gene. H gene tree also displays this new strain of CDV as a separate clade (blue) from previously identified genotypes, including the America 2 genotype (red clade). Based on H gene sequence analysis, there are at least three separate “American” clades of CDV currently circulating in the U.S

Both CDV 13–1941 and 13–2262 were sequenced to near completion including all coding sequence and intergenic space and sequences were deposited [GenBank: KJ747371 and KJ747372], respectively. Reference complete or near-complete genomes from GenBank were aligned to our two sequences and used to develop a phylogenetic tree (Fig. 2). Hemagglutinin (H) gene sequences from these samples were aligned with H gene sequences in GenBank and were also used to develop a phylogenetic tree (Fig. 3). Phylogenetic trees based on individually extracted protein sequences for 6 genes (N, P, M, F, H, L) in the genome displayed similar relationships to the whole genome analysis and thus were not included in this manuscript. CDV 13–1941 and CDV 13–2262 outgroup in relation to the other genome sequences and form a distinct clade with extremely limited heterogeneity. A comparison of amino acid differences among hemagglutinin genes (commonly used to investigate phylogenetic relationships and evolution of CDV) [3] from different lineages supports this relationship (Table 1).

**Table 1:** H gene divergence/percent identity

AS2	88.74											
EU/SAM1	89.24	92.26										
AFRICA	89.90	93.08	94.23									
AS1	89.40	93.25	92.75	93.57								
13_1941	89.40	92.74	93.40	93.40	93.56							
13_2262	89.40	92.75	93.41	93.41	93.57	99.67						
SAM3	89.07	92.26	91.93	92.75	92.59	93.56	93.57					
EW	90.07	93.09	93.25	93.75	93.58	94.56	94.57	93.42				
19876	90.23	93.25	93.57	94.23	94.07	94.55	94.56	93.90	94.41			
SAM2	89.74	92.26	92.92	93.25	93.41	94.22	94.23	93.25	94.41	94.40		
AM2	90.89	93.41	94.07	94.73	95.06	95.05	95.06	94.73	95.39	95.39	95.22	
EU	90.73	93.90	94.23	94.40	95.06	95.21	95.22	95.22	95.56	95.72	96.05	96.87
	AM1	AS2	EU/SAM1	AFRICA	AS1	13_1941	13_2262	SAM3	EW	19876	SAM2	AM2

GenBank numbers: AF378705 (AM1, America 1); Z54166 (AM2, America 2); AB687720 (AS1, Asia 1); AB474397 (AS2, Asia 2); DQ228166 (EW, European Wildlife); FJ461723 (AFRICA); KC966929 (ARCTIC); DQ494319 (EU, Europe); Z47761 (EU/SAM1, Europe/South America 1); FJ392651 (SAM 2, South America 2); KF835420 (SAM3, South America 3); AY964110 (19876- USA)

**Fig. 4:**
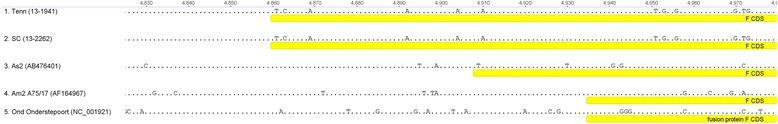
Alternate start sites for fusion (F) gene. The new strain (top two sequences) has a predicted start site 75 bp preceding that for America-2 A75/17 and Onderstepoort and 27 bp from Asia-2. This adds 25 and 9 amino acids, respectively, to the signal peptide region of the protein

In addition to strong sequence conservation in the new genotype, genome sequencing revealed an alternate start codon for both sequences (13–1941 and 13–2262) of the fusion (F) protein, adding 75 nucleic acids (25 amino acids) to the signal peptide region relative to its closest relatives on the phylogenetic tree (Fig. 4). Evaluation of the hemagglutinin protein showed this strain has 7 potential N-glycosylation sites (N149, N309, N391, N422, N456, N587, N603). It lacks the site at N584 that has been found in some Asian strains [14].

**Fig. 5:**
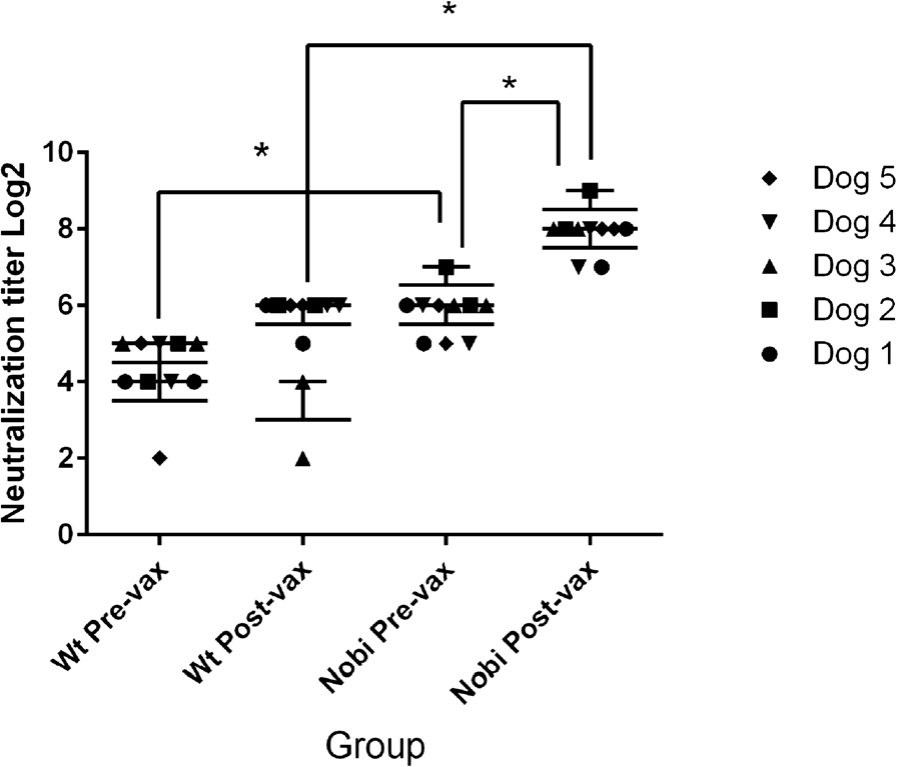
Serum neutralization assay. Five adult research dogs were vaccinated with a modified live virus vaccine approximately 1 year after vaccination with the same vaccine. CDV titers were determined by serum neutralization using either the new strain or the vaccine strain (Nobi). Viruses were each used at 100 TCID_50_ per well and the results are reported as the means of three replicates. The experiments were repeated, and the results for both experiments are included. The titers were not significantly different between pre-vaccination and post-vaccination when evaluating titers with the new strain. There was a significant difference between pre- and post-vaccination titers when testing with the vaccine strain, suggesting the memory response was specific for the vaccine virus

Serum obtained from 5 adult dogs prior to and following a yearly booster vaccination with a modified-live CDV vaccine showed statistical differences in serum neutralization titers when the vaccine strain virus was used in testing versus a clinical isolate of the new strain. Pre-vaccination geometric mean titers for the vaccine strain and the new genotype were 42.09 (low titer 32, high 128) and 21.11 (low titer 8, high 32), respectively. Post-vaccination geometric mean titers for the vaccine strain and the new strain were 240.37 (low titer 128, high 512) and 52.05 (low titer 8, high 64), respectively. There was a statistically significant difference (*p* = 0.05) between titers pre- and post- vaccination against the vaccine strain but not between titers pre- and post-vaccination against the new strain. There was a statistically significant difference (*p* = 0.05) between the titers both pre-vaccination and post-vaccination when comparing the titers between the two viruses (Fig. 5).

## Discussion

This new strain of CDV is highly conserved over the period of surveillance, geographic region and host species. It appears to be widespread in the Southeast US, but samples evaluated were samples of convenience (those submitted to the diagnostic lab for CDV testing). Additional states are likely involved but more samples are required from both healthy and symptomatic animals for an epidemiological survey.

Unlike a previous report that suggests strains stemming from the America-2 lineage are the dominant “American” strain in circulation [15], at least in eastern Tennessee, this new strain was predominant in 2011–2013. We consider this to be the third “contemporary” genotype that has been detected in North America.

The first contemporary genotype described was America 2 (represented by red clade, Fig. 3). The term genotype has been used in the grouping of CDVs, but a formal analysis has yet to be published. Genotypes have been defined by the phylogenetic properties of the H-gene amino acid sequence. Strains in the same clade showing more than 95 % amino acid homology are considered to belong to the same genotype [14]. This is an arbitrary distinction, and depending on which sequences are used for comparison the 95 % difference does not always hold true when considering currently recognized genotypes. This is evident when comparing one of the European strains (GenBank Accession DQ494319) with a European Wildlife strain (GenBank Accession (DQ228166) versus the same comparison when using a different European strain (GenBank Accession Z47761) (Table 1).

**Table 2:** Primer sets for genome amplification

Set	Forward	Reverse	Nucleotide position^**a**^
F1/R1	GAGAACAAGGTCAGGGTTCAG	TTGCCGGCAGATCTTCTAAC	77–1231
F2/R2	TGCTATGGGAGTTGGTGTTG	TCTTCGCCAGAATCCTCAGT	1112–2273
F3/R3	CGAAGATGCTGACAGTCTCG	GAAAGCAGTTCTGTGCCTGTT	2169–3368
F4/R4	CAAAGTCACAAACACAACATGC	GGGACTGATGGTTGCAAGAC	3223–4372
F5/R5	TGTTACCCGCTCATGGAGAT	CCAAGTACTGGTGACTGGGTCT	4272–5433
F6/R6	GGTGCATTGGAATAGCCAGT	GCAGGTATCGGAGGCAATAA	5299–6530
F7/R7	CTTGGTGTCTGGGACGATG	GCTGCCGATGCAATAGATTT	6377–7578
F8/R8	GGTTACGGTTGCCACAAAAA	TCCATAATCTGGGATGTTTGAA	7389–8580
F9/R9	AAGAACGGAACAGTCCTTGG	TTGTGACTGGTGAGGTCAATG	8441–9685
F10/R10	TGCAAAGCTCACAGTGGTTC	CAGGCTCGCATTTTGTAGGT	9496–10696
F11/R11	GAGGACTCTCAGTTTGACCCTTA	CACATCCCGTGTCATAGCTG	10539–11777
F12/R12	CCGCATGCAGTAACATTTCA	GCCCATGAGTACACAGTTGCT	11626–12772
F13/R13	TACATCGGGTCCACAACAGA	CCTCATCACTTTCGCACAAA	12663–13821
F14/R14	CACGGACCCTCTCTTGACTC	CGAGGTAGGCCTCTGTTGAC	13683–14901
F15/R15	GCGACTGGGTTCAAGGATTTA	AGCAATGAATAGCAGAGGGTTAG	14797–15623

^a^Reference genome AF164967 (A75/17)

We performed phylogenetic evaluation of current US strains in comparison to our strains and discovered circulation of another clade that has already been described but not well-defined in the literature (yellow clade, Fig. 3). A virus belonging to this clade was initially detected by Pardo et al. in 2005 [16], sample 19876 (GenBank accession AY964110), detected in Missouri, USA in 2004 but was not defined as a new genotype at that time. Strains belonging to the clade represented by sample 19876 were also detected by additional researchers and suggested to be part of the European Wildlife lineage [5] or a subgroup thereof [17] (CDV outbreak in fishers in California (GenBank accession numbers JN836734-7), based on partial H gene sequencing. Strains grouping with the 19876 clade were later also detected in Mexico (Edomex strains) and the researchers defined the clade as a new genotype/lineage [4]. Based on our phylogenetic analysis, we concur the clade represented by sample 19876 should be recognized as a new genotype based on H gene divergence (Table 1) and viruses from this genotype are currently circulating in Tennessee and Texas in the US.). Therefore, in staying with the current genotype naming scheme, this clade represents America 3 (yellow clade, Fig. 3).

Based on clade groupings and hemagglutinin amino acid divergence, we suggest this new strain circulating in the Southeast also represents a new genotype and should be called America 4 (blue clade, Fig. 3). This new America 4 genotype outgroups from other clades with similar distances as each previously described genotype does from each other. SNPs between the clades outnumber SNPs within each clade, yet CDV 13–1941 and 13–2262 have fewer differences between them than all but clonally derived isolates (only 56 nucleotide differences across the two genomes). This is unexpected as these two samples came from different species of animals, at different times and in different location with no obvious route of common exposure or transmission. It is also highly conserved, supporting the hypothesis that this novel lineage is most likely contained in a stable host reservoir with breakthrough transmission to other species.

In addition to the sequence divergence from other clades, an alternate start site increasing the size of the signal peptide of the F protein has been previously reported in the Asia-2 lineage [6]; however, this new strain adds 9 additional amino acids even beyond the Asia-2 variant. It has been suggested that variations in the signal peptide region affect F protein expression and a longer signal peptide region might result in reduced F protein production and persistent infection due to evasion of the immune system [18, 19]. This observation, in addition to the highly conserved genome, fits with our hypothesis that this strain is representative of a stable reservoir. Figure 1 reveals that the hypervariable M-F region is practically identical, if not completely identical in most of the isolates typed as the new strain. This means that the two genomes we sequenced are unlikely to be coincidentally conserved and the other isolates are, in all probability, equally conserved at the genomic level.

A virus capable of infecting and transmitting in wildlife yet not causing severe disease in a large number of infected animals would be under less selective pressure and show reduced genomic divergence between isolates, as we observed in our data. Furthermore, if this strain is significantly divergent from vaccine strains (which is also shown in our data/Figures), it may be capable of vaccine escape which is supported by our data from vaccinated dogs and our serum neutralization testing. The exact mechanism of vaccine protection in dogs with respect to CDV is unknown. Therefore we do not know what mutations, or combination of mutations, give rise to vaccine escape and host adaptation for this strain. However, if it is well-adapted in wildlife, it follows that infection in domestic dogs would be inefficient and/or infrequent. Why exactly this strain is only recently causing disease in dogs if it has been in wildlife long enough to become a reservoir is unknown; it may have been around prior to our discoveries yet undetected due to lack of genetic evaluation of strains. However, the lack of diversity in a hypervariable region from multiple dogs suggests this is a newly introduced strain.

We believe the stable host reservoir is likely the endemic wildlife population. The virus is highly labile outside of the host, so cross-species transmission occasionally occurs, thus the virus could be maintained in wildlife populations between outbreaks [20]. We have detected this strain in raccoons and foxes, as well as three dogs with a history of recent direct raccoon exposure. Raccoons, which are among the most common wildlife species found in cities and towns, are considered a secondary reservoir of CDV and spillover of infection from domestic dogs with spillback from raccoons is well documented [1].

While there have recently been multiple reports of new genotypes/lineages, questions remain as to whether the genetic differences represent significant differences in antigenicity. It has been suggested that changes in the glycan shield may result in vaccine escape mechanisms. However, comparative neutralization studies have shown that fully glycosylated and de-glycosylated wild-type viruses are neutralized with equal efficiency [21]. It was also demonstrated that only 5 of the N-glycosylation sites (N149, N391, N422, N456, and N587) are actually used by the virus and this glycosylation pattern has been highly conserved in wild-type strains. This argues against a glycan shield escape mechanism for CDV, and the presence of N309 detected in this new strain and in other recent wild-type strains, as well as N584 in some Asian strains, is meaningless [21].

CDV is currently recognized as a single serotype [22]. However, preliminary evaluation of neutralization titers comparing this strain to one of the America 1 type vaccine strains showed significant differences (Fig. 5). Neutralizing antibodies are often used as a substitute marker for protection [23], with a neutralization titer of ≥ 32 generally considered protective [24]. Evaluation of the titers from this study, in light of this cut-off value, suggests all 5 dogs would not have been considered protected pre-booster, and one dog would not have been considered protected post-vaccination when evaluating neutralization titers with this new strain. All the dogs would have been considered to have a protective titer both pre- and post-vaccination when evaluating the antibody response with the Onderstepoort vaccine strain. These results should be considered from a diagnostic standpoint with regard to which virus strain or strains should be used for routine serum neutralization testing for dogs.

The extent to which antigenic variation is leading to vaccine failure is unknown. This new strain has been associated with disease in 3 properly vaccinated adult dogs. All three dogs were euthanized due to severe clinical disease. Two of the dogs were necropsied and histologic findings were consistent with CDV infection. Unfortunately, sera from these dogs were not available for antibody testing. While it is possible these dogs were vaccine non-responders, it would not be expected based on the percentage of these animals seen in the general population (approximately 1/5000 dogs, http://www.wsava.org/sites/default/files/WSAVA_OwnerGuidelines_September2010.pdf), and the fact that these dogs were all different breeds and unrelated.

Cell-mediated immunity must also be considered with regard to vaccine protection. This was not evaluated in this study, but presence of a neutralizing titer correlates with the presence of a T-cell response, though they are not well correlated quantitatively [23]. A protective cellular immunity can be present in the absence of a detectable antibody-mediated response. A strong cellular immunity causes viral elimination but a delayed or absent lymphocyte-mediated response results in viral persistence in the CNS [25]. Challenge studies with this new strain would best determine whether dogs are protected from the new strain via cross-protection or if the new strain is genetically distinct enough to evade the vaccine but that was beyond the scope of this project.

Critical CDV-specific herd immunity resulting from low vaccination rates may contribute to outbreaks [22]. Vaccine histories were unknown for some of these animals, but the majority of the cases were in dogs with incomplete vaccination or no vaccination. This suggests herd immunity is low due to reduced vaccination rates. This has likely contributed to the emergence of this new strain. Whether there is also reduced protection in vaccinated dogs as a result of extended vaccination intervals is unknown. The issue of duration of protective immunity maintained by booster vaccination every 3 years for CDV may have to be reevaluated in light of the emergence of new variants of CDV [5]. The veterinary profession should weigh the risks of side-effects due to over-vaccination against the risk of a decreased herd immunity with the re-emergence of epidemics as a consequence [26]. Maintenance of high vaccination rates using efficacious vaccines that induce a solid, resilient immunity must still be given the highest priority in control of distemper [22]. Our data suggests that ongoing surveillance should be conducted to detect and analyze new and emerging strains of CDV that may facilitate vaccine evasion.

## Conclusion

There is a newly detected lineage of CDV circulating in the Southeastern portion of the United States. It is genetically distinct from all previously identified genotypes, yet highly conserved within its own lineage. Continued and broadened surveillance, including wildlife, will determine the impact of this genotype in comparison to other circulating genotypes. While there have been vaccine breaks associated with this genotype, vaccination remains the best way to manage CDV in the absence of a specific treatment for this virus.

## Methods

Samples were submitted between 2010 and 2014 to the UTCVM Clinical Virology Lab for canine distemper virus detection. Submissions were mainly canine from eastern Tennessee, but samples from Canada, Texas, Washington, Kentucky, West Virginia, Virginia, and South Carolina were also tested. Samples were also submitted for wildlife including raccoons and foxes from Tennessee.

RNA was extracted from clinical samples (including nasopharyngeal/conjunctival swabs, urine, and tissues) and cell culture supernatant containing virus with a commercially available extraction kit (QIAamp Viral RNA Mini Kit, Qiagen, Valencia, CA, USA) as previously described [7]. RNA previously extracted was stored at −80 °C.

Samples were tested for CDV by real-time RT-PCR. cDNA from positive samples were genotyped by sequencing the variable M-F intergenic region, and a phylogenetic tree was constructed according to previously described methods [7].

Samples used for genome sequencing included CDV sample number 13–1941, which was isolated from lung that had been stored at −80 °C from a fox that displayed neurologic signs prior to euthanasia and tested negative for Rabies virus. It was submitted to the pathology department for disposal from the Avian and Zoological Medicine service in the UTCVM. The virus was cultured on Vero SLAM cells (kindly provided by Dr. Edward Dubovi, Cornell University, NY, USA). CDV sample number 13–2262 was collected via urine from a 6 month old puppy from South Carolina with clinical signs consistent with canine distemper, and the sample had a real-time RT-PCR Ct value of 13.11. This strain was not isolated and whole RNA from the urine sample was used directly for genome amplification.

Approximately 50 bp overlapping primer sets with ~1000 bp products were designed using Primer 3 [27, 28] to amplify complete CDV genome sequence (minus the extreme 5’ and 3’ non-coding ends) (Table 2). Two μL of RNA per sample were run in 25 μL total volume reactions using a commercially available master mix (SuperScript III Platinum One-Step RT-PCR kit, Invitrogen, Life Technologies, Grand Island, NY, USA) using 300 nM of each primer and one unit of RNAse inhibitor (RNAse Out, Invitrogen, Life Technologies, Grand Island, NY, USA) for RT-PCR. Samples were amplified in a thermal cycler (GenePro, BIOER Technology, China) with a RT step at 50 °C for 30 min., activation step for the hot start Taq polymerase at 94 °C for 2 min., followed by 35 cycles of denaturation at 94 °C for 30 s., annealing at 60 °C for 1 min., and elongation at 72 °C for 3 min., with an additional elongation step at 72 °C for 10 min. The RT-PCR products were electrophoresed on a 1 % TBE agarose gel stained with SYBR Safe®, and visualized by SYBR© Green-filtered UV light with a CCD camera system (UVP, Inc., Upland, CA, USA). Products with a single band were purified using ExoSAP IT (Affymetrix, Santa Clara, CA, USA). Products with more than one band but a single clear product at ~1000 bp (3/15 sets) were excised and gel purified (QIAquick gel extraction kit, Qiagen, Valencia, CA, USA) and all products were capillary sequenced at the UT Molecular Biology Core Facility using the same primers used for the PCR reactions.

Chromatograms for capillary DNA sequence were manually edited and assembled using Geneious©, and all positions in the sequence had at least 2x coverage. Available reference genomes and H genes representing the major CDV lineages were downloaded from GenBank and aligned to CDV 13–1941 and CDV 13–2262 using MAFFT v7.017 [29]. Nucleotide substitution model GTR was selected using ModelGenerator v0.85 [30] and phylogenetic trees were constructed using MRBAYES v2.0.9 (genomes) [31] with 1,000,000 iterations with subsampling every 1000 trees and a burnin of 20,000 iterations [32] or Neighbor-Joining method (H gene). Individual coding sequences (CDS) were predicted using GLIMMER3 v1.4 [33], extracted, translated and aligned to representative sequences from GenBank. Phylogenetic trees for individual protein sequences were generated using UPGMA in Geneious© Tree Builder (version 6.1.4, Biomatters http://www.geneious.com) with the Jukes-Cantor distance model.

For evaluation of antigenicity, blood was collected from 5 adult dogs that are part of the UTCVM teaching colony, according to Institutional Animal Care and Use Committee guidelines (University of Tennessee Knoxville IACUC protocol 1954). Samples were collected from the animals just prior to receiving a Nobivac vaccine (Merck Animal Health, Summit, NJ, USA) and again 5 days following vaccination. Each dog had been given the same vaccine approximately 1 year prior. Serum was separated from the cells and stored at −20 °C. Neutralization titers were determined by a standard procedure [34] using 100 TCID_50_ per well of the fox isolate (13–1941) or Nobivac isolate. The Nobivac isolate was prepared in the same manner as described for the fox isolate. Samples were run in triplicate and each test was duplicated to evaluate reproducibility. A Mixed ANOVA method (SPSS Software, SPSS Inc, Chicago, IL, USA) was used to compare serum neutralization titers between the viruses and the two time points. In order to determine which pairs of means were significantly different, mean separation tests were run using the Tukey-Kramer method at a significance level of *p* = 0.05.
